# Teaching home tonometry using a remote video link

**DOI:** 10.1038/s41433-022-01966-y

**Published:** 2022-02-17

**Authors:** Catriona C. Barbour-Hastie, Andrew J. Tatham

**Affiliations:** 1 University of Edinburgh, Edinburgh, UK; 2 Princess Alexandra Eye Pavilion and Centre for Clinical Brain Sciences, University of Edinburgh, Edinburgh, UK

**Keywords:** Health services, Technology, Outcomes research

## Abstract

**Background/Objectives:**

Intraocular pressure (IOP) is the primary modifiable risk factor in the progression of glaucoma. The ICare HOME is a self-tonometer which empowers patients to measure their own IOP and allows a more complete picture of diurnal IOP. This project aims to determine the feasibility of teaching patients to perform self-tonometry remotely using a remote video link.

**Subjects/Methods:**

This prospective study involved 12 patients with glaucoma attending an outpatient ophthalmology clinic. Participants were provided with a rebound tonometer (Icare HOME) and instructions to attend remote teaching from home. An optometrist conducted a 30 min live video training session via NearMe with each patient. Following training, participants were asked to measure their own IOP, observed remotely by the optometrist. Successful participants were asked to take a series of home IOP measurements over 48 h. Questionnaires were used to evaluate perceptions on home tonometry and remote training.

**Results:**

Participants had an average age of 60.1 ± 15.5 years. 58% (7 of 12) were female. 83% (10 of 12) obtained successful diurnal measurements at home. All participants were happy with remote teaching, and none would have preferred training to be conducted face-to-face. All participants were interested in continuing home IOP monitoring.

**Conclusions:**

Most patients were able to perform home tonometry successfully when taught remotely, with a success rate similar to previously reported rates for face-to-face teaching using the same device. Most participants were receptive to using video calling as a platform for teaching home tonometry.

## Introduction

Glaucoma is a leading cause of preventable sight loss and thus a major public health concern [1]. Raised intraocular pressure (IOP) is the primary modifiable risk factor for the development and progression of glaucoma and current treatments target IOP reduction for visual field preservation. Accurate measurement of IOP is therefore essential for glaucoma risk stratification and for determining therapeutic effect. The accepted standard for IOP measurement is Goldmann applanation tonometry (GAT), however GAT must be performed in-person by a trained healthcare professional and therefore using GAT, it is only possible to obtain infrequent IOP measurements. IOP fluctuates, and there is increasing evidence that the magnitude of fluctuation may be an important risk factor for progression [2].

To obtain a more complete picture of IOP fluctuation, and more precisely determine therapeutic effect, IOP can be measured throughout the day in the office or patients can be admitted to hospital for repeated GAT. However, obtaining repeated office-based diurnal IOP measurements is costly, inconvenient, and time consuming. Home tonometry offers a potential alternative, particularly attractive since the SARS-CoV-2 pandemic, with the increased need to undertake healthcare appointments remotely. Several studies have reported positive findings on ease of use and reliability of Icare HOME (TAO22; Icare Finland Oy, Vantaa, Finland), a rebound tonometer (RT) designed for self-tonometry. Our group has previously demonstrated that self-tonometry is feasible, with the majority of glaucoma patients able to successfully measure their own IOP using the RT after a 30 min face to face training session, and several studies have demonstrated high repeatability of home RT measurements.

Though the Icare HOME enables patients to measure their own IOP at home, previous studies of the device have relied on patients attending face to face training in the hospital or office setting. Remote training would offer the possibility of true remote monitoring and be particularly advantageous for patients living a long distance from their healthcare provider, or when patients are unable to attend for other reasons. Tonometers could be mailed to patients for use during the monitoring period and returned to the health provider on completion. To the best of our knowledge, no previous studies have evaluated the feasibility of teaching home tonometry remotely. The purpose of the current study was to establish whether patients with glaucoma can be taught self-tonometry remotely and determine if they can continue to take reliable measurements in their own home.

## Methods

This was a prospective service evaluation involving 12 patients with glaucoma recruited from the glaucoma clinic at the Princess Alexandra Eye Pavilion, Edinburgh, Scotland. The project was prospectively approved by the Princess Alexandra Eye Pavilion quality improvement committee.

All participants had a comprehensive eye examination, including assessment of visual acuity, slit-lamp examination of the anterior segment, gonioscopy, IOP assessment using Goldmann applanation tonometry, measurement of central corneal thickness, dilated fundoscopy, visual field assessment using standard automated perimetry (SAP, SITA Fast 24-2 test using the Humphrey Visual Field Analyzer, Zeiss), and optical coherence tomography (OCT) of the retinal nerve fibre layer (RNFL). The diagnosis of glaucoma was made by the treating clinician. Potential participants were asked whether they had access to the internet and to a device that enabled online video calling, such as a personal computer, tablet computer, or smart phone. Those without access to such a device were excluded. Potential participants who had previously performed self-tonometry were also excluded. We also excluded patients with a recent history of intraocular surgery (within 3 months).

Participants were given the Icare HOME device and a patient information leaflet with written instructions on how to use the device. An online appointment was scheduled for remote training using Near Me. Near Me is a secure online video consulting service launched in 2016 to meet the demand from rural Scotland to reduce the need to travel for healthcare appointments [3]. Participants were not provided with instructions on how to use the device at the clinic appointment and were instructed to wait until the remote consultation before attempting to use the tonometer.

All remote teaching sessions were conducted by the same investigator with the teaching portion of the video call lasting a maximum of 30 min. The training was stopped once the investigator was happy with the patient’s technique, with the time required to achieve this recorded. Participants were then asked to use the device to measure their own IOP 8 times over two consecutive days; four times daily, spaced approximately four hours apart. The Icare HOME determines IOP based on rapid collection of 6 consecutive readings, discarding the highest and lowest readings, and recording the average of the remaining 4 measurements. The date and time of each measurement, identification of right and left eye and the quality of the measurement based on the standard deviation of the remaining 4 measurements are recorded automatically and once the device was returned, the data was transferred to a personal computer for analysis. Participants were deemed to have successfully learnt to use the device if the following criteria were met:

1. The positioning of the tonometer was correct during measurement, determined by the investigator observing the patient during the video training call.
2. The patient was able to take 3 reliable self-measurements by the conclusion of the training call.
3. The participant was able to obtain a minimum of 7 of 8 measurements, during the two-day monitoring period, which were deemed good quality by the Icare HOME.

Some previous studies have included a requirement for IOP measurements using Icare HOME to be within 5 mmHg of GAT, as part of the successful measurement criteria. All participants in the current study had their IOP measured by GAT as part of their outpatient appointment, however, due to the tutorial being conducted remotely on a different day and often at a different time to their outpatient appointment, and due to the fluctuating nature of IOP, it was decided not to include a comparison to GAT as a success criterion.

Participants were also asked to respond to 6 statements about their perception of ease of use of the Icare HOME and their experience of the video call teaching session. Each statement used a 5-point Likert scale to gauge agreement or disagreement. The statements included:

1. In your opinion, you are able to take successful measurements.
2. The Icare HOME is easy to use.
3. The measurements are comfortable to take.
4. You would be happy to use the Icare HOME in the future.
5. You were happy to be taught remotely.
6. It was easy to learn how to use the Icare HOME via video call.

Two yes/no questions were also asked; would you have preferred to have had a face-to-face appointment to learn how to use the device? And, if no, would you have answered differently before the pandemic? Questions 2–4 correspond to questions asked in a study evaluating the ability of patients with glaucoma to perform self-tonometry after face-to-face teaching [4]. A short unstructured interview followed where comments and feedback were typed up verbatim in a results table for qualitative analysis.

## Results

The demographic and clinical characteristics of the group are displayed in Table 1. Participants had an average age of 60.1 ± 15.5 years. 58% (7 of 12) were female. SAP mean deviation (MD) in the worse eye was −6.03 ± 8.57 dB and −1.82 ± 5.11 dB in the better eye. Mean IOP with GAT was 19.1 ± 6.6 mmHg (range 12–32 mmHg). Participants were using an average of 1.6 ± 0.9 eye drops (range 0 to 3). 83% (10 of 12) completed the training successfully and were able to obtain IOP measurements at home consistently for 2 days. Participants who were successfully able to perform home tonometry required a training session with an average duration of 15.2 min. There were no technical difficulties encountered that interrupted the tutorials.

**Table 1:** Demographics and clinical characteristics of participants.

Variable		Mean (SD)	Range			Median	Quartile 1	Quartile 3
Age (years)	All	60.1 (15.5)	25	to	76	65.5	52.25	69.75
	Male (42%)	60.8 (86)	50	to	68	66	53	67
	Female (58%)	59.6 (19.8)	25	to	76	65	50	75.5
Visual acuity (logMAR)	Better eye	−0.01 (0.14)	0.3	to	−0.2	0	−0.1	0
	Worse eye	0.11 (0.24)	0.6	to	−0.1	0	−0.03	0.23
SAP, mean deviation (dB)	Better eye	−1.82 (5.11)	−17.53	to	1.36	−0.42	−1.86	0.57
	Worse eye	−6.03 (8.57)	−27.73	to	0.24	−2.87	−7.69	−0.4
Central corneal thickness (micrometres, μm)		567.5 (54.9)	501	to	683	553	528	606
IOP by GAT in clinic (mmHg) *n* = 24 eyes		19.1 (6.6)	12	to	32	16	14.75	24.3
Number of eyedrops		1.58	0	to	3	1.5	1	2

*SAP* standard automated perimetry, *GAT* Goldmann applanation tonometry.

2 of 12 participants (16.7%) were deemed unsuccessful; one of whom was unable to obtain any IOP measurements during the training session. This patient was recalled for face-to-face training but was still unable to measure their own IOP successfully. The second unsuccessful patient managed to obtain a single IOP measurement during the teaching session but on analysis of IOP readings taken over the subsequent 2 days, only 4 of 16 measurements taken were successful and these were classed as poor quality by the device.

**Fig. 1:**
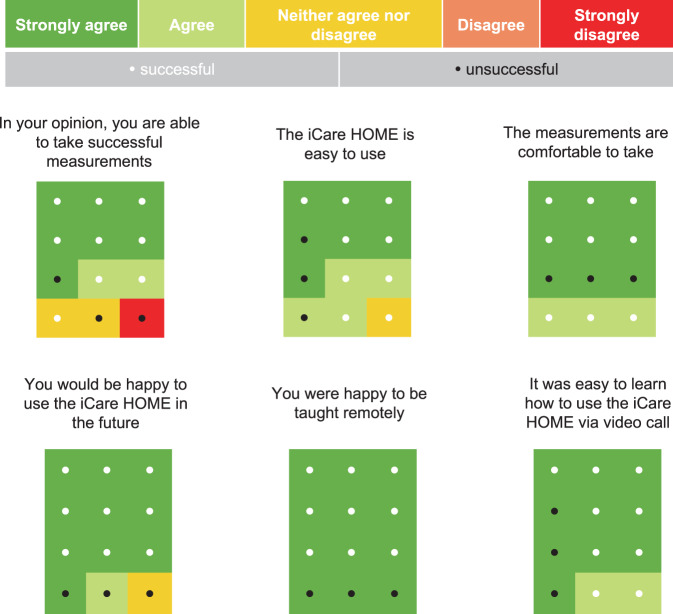
Visualisation of responses to the 6 Likert scale items. One large rectangle represents 100% of participants (*n* = 12).

Figure 1 provides a summary of responses to the six, 5-part Likert statements. 9 of 12 participants (75.0%) agreed or strongly agreed with the statement “in your opinion, you are able to take successful measurements”, with only one person disagreeing. No participants disagreed with the statement “The Icare HOME is easy to use” and all either agreed or strongly agreed that measurements were comfortable to take. 11 of 12 participants agreed or strongly agreed that they would be happy to use to Icare HOME in the future. For the two statements specifically addressing the remote teaching, all participants strongly agreed that they were happy to be taught remotely, and that it was easy to learn how to use the device via the video call.

**Fig. 2:**
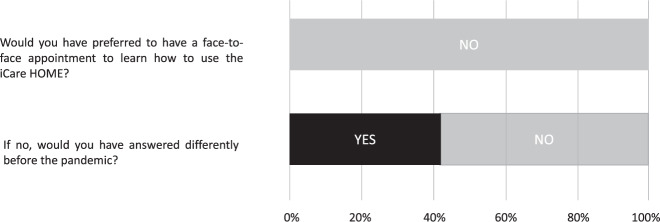
Visualisation of responses to the two yes/no questions. Stacked bar chart displaying responses to yes/no questions from all 12 participants (100%).

When asked whether they would have preferred to have a face-to-face appointment to learn how to use the device, no participants replied that they would. However, when asked whether they would have responded differently before the covid pandemic, 5 of 12 (41.7%) responded that they would have preferred a face-to-face appointment (Fig. 2).

The qualitative interview revealed some common themes. 11 of 12 participants (91.7%) were using at least one anti-glaucoma topical medication daily and some suggested that this facilitated their success in performing self-tonometry (Quote 1).

Quote 1 “*Maybe people who haven’t been used to being near their eyes, with eyedrops and all the things I’m used to, might find it more difficult*.”

A similar perception was proposed by two participants who were regular contact lens wearers. Despite the device using disposable probes and being disinfected before being given to participants, the anxiety about COVID transmission by fomites was highlighted by one participant (Quote 2).

Quote 2 “*I’m sure some people might want some reassurance that the machine hasn’t got COVID on it, you know*.”

The Icare HOME does not show the user their IOP, and this feature was a common, unprompted theme that seemed to divide participants (Quotes 3 and 4).

Quote 3 “*I’m not interested in knowing my pressures thanks, I just think it would make me neurotic about them. I prefer just not knowing. I trust the doctors to tell me if there is a problem that needs addressed*.”

Quote 4 “*It’s a shame I can’t see the pressures myself, I think it would reassure me to know what they are. Can you let me know what they are?*”

Several advantages of learning virtually were offered by patients including travel time saved (Quote 5) along with confidence in their ability to use the machine on their own (Quote 6).

Quote 5 “*I think now I would prefer learn like this. It saves me time and also saves transport as I can’t drive anymore and parking [at the hospital] is a nightmare for my husband*.”

Quote 6 “*It’s probably actually better to learn at home instead of being in the hospital as you have more confidence you can actually do it at home yourself; because you’ve already done it*.”

Anxiety about COVID infection for face-to-face appointments was apparent in at least 2 patients and so virtual teaching was preferable (Quote 9).

Quote 9 “*Since lockdown I have had problems with anxiety about the virus, so I try to avoid interactions in person, especially in hospitals*”.

English was a second language for 2 patients in the project, where unforeseen advantages about time to understand and translate what is being asked of them, were revealed. (Quote 7)

Quote 7 “*I like video because before the call I have time to translate the email and [patient information leaflet]. I can understand better*.”

The increased familiarity with online video calling since the pandemic was described frequently, particularly in the older age groups (Quote 8).

Quote 8 “*Before the lockdown, I had never used video calls before, but now I play online bridge and “Zoom” my family frequently, so I’m much more comfortable with video calls now*.”

## Discussion

To the best of our knowledge, this is the first study examining the feasibility of remote teaching of home tonometry. Overall, a similarly high proportion of patients with glaucoma were able to successfully measure their own IOP as reported in previous studies where self-tonometry has been taught face to face [4–6]. The results also indicate that patients are happy to be taught remotely, further suggesting that this is a feasible option for teaching self-tonometry. Also in agreement with previous studies, the majority of patients found the home rebound tonometer comfortable to use and were interested in performing home IOP monitoring in the future [4, 7]. All participants agreed that it was easy to learn self-tonometry via video call, across all ages and including those whose first language was not English. Surprisingly, even the two patients who were unable to perform self-tonometry successfully expressed an opinion that self-tonometry was easy to learn by video call, which on further questioning was due to their perception that no matter the format of teaching, they felt they would struggle to use a device to measure their own pressure.

Remote teaching has some potential advantages over face-to-face teaching, including that the patient does not necessarily need to attend the clinic to learn how to use the device, and if needed further calls could be arranged to check progress. As IOP measurements obtained using the Icare HOME can be uploaded to the cloud and remotely accessed by the clinician, remote video calls could also be used to provide feedback to patients on their IOP measurements. It is possible that patients may be able to use the device successfully without either face to face training or a video call, for example, by viewing a training video, or reading the instruction booklet however, by using a video call, the clinician can visually evaluate and suggest adjustments to the device and patient technique.

This small but typical group of glaucoma patients were receptive to using video calling as a platform for healthcare interactions and of using home tonometry to provide additional information to monitor their glaucoma. A large proportion of participants expressed the view that they would have opted for a face-to-face appointment had it not been for the COVID pandemic. This appeared to be due to greater familiarity with the use of video healthcare consultations in general, which have increased exponentially since the pandemic. In Scotland, there were almost 17,000 Near Me consultations in the last week of June 2020, contrasted with only 300 consultations per week in February 2020 [3]. Similarly, online communication services for personal use more than doubled, where video calling (at least weekly) rose from 35% in February 2020 to 71% in May 2020. This growth trend was particularly evident among adults aged 65 and over whose use of video calling at least weekly increased almost threefold, from 22% in February 2020 to 61% in May 2020 [8]. The pandemic seems to have created an opportunity that has led to fundamental behaviour changes.

Disadvantages of teaching using telemedicine, include exclusion of patients who lack internet access or suitable devices for internet video calls, and the likelihood that the older age group, typical of those with glaucoma, may be less able to use the technology. However, in 2020, 96% of UK households had access to the internet, a figure continues to rise year on year with 77% of adults aged 65 and over having accessed the internet at least weekly, and only 5% of UK adults having not accessed the internet in the last 3 months [9].

The pandemic has created complex challenges around the delivery of glaucoma care, with new risk stratification models developed to help weigh up risk of lifetime sight loss with risk of harm from COVID-19 infection [10]. We identified that patient attitudes may also shifted, where some remain eager to use video calls for healthcare interactions where possible, due to fear of the virus and to minimise risk of transmission. Perceived threat is positively associated with preventative behaviour [11], but delay or avoidance in accessing healthcare due to the pandemic may have long-term impacts for patients, especially those in poor health [12]. Teaching home tonometry remotely offers the possibility of a remote IOP monitoring service for glaucoma patients, which may prove valuable in conjunction with novel portable home-monitoring automated perimetry, which is on the horizon [13, 14].

The study has some limitations, including the small sample size, and the short duration of home IOP monitoring. Furthermore, though patients were recruited consecutively from clinic, sample selection bias was present as only patients who had the facility for video calling were included. Although consecutive patients were asked to participate in the project, those who declined to participate were not consistently documented nor interviewed to explore their decision therefore we are unable to identify the demographic of glaucoma patients that would not be happy to or might be unable to learn self-tonometry remotely. Allowing patients to self-teach the Icare HOME for example, via a YouTube link, could also be explored as this would further minimise reliance on resources such as staff time, however, this is contraindicated by the manufacturer in the instruction manual which states that “all patients must receive training from a certified healthcare professional prior to performing self-tonometry”.

As home tonometry consistently attests to being reliable and acceptable to patients with glaucoma, future research involving a larger cohort will be useful. It will also be important to determine which subgroups of patients are most likely to benefit from home monitoring and whether the greater number of IOP measurements obtained with home monitoring improve the ability to predict risk of progression, or measure responses to treatment. Nevertheless, this study indicates that home tonometry can be taught remotely, and that patients with glaucoma receptive to using video calling as a platform for healthcare interactions.

### SUMMARY

#### What was known before

- Home tonometry empowers patients to measure their own intraocular pressure (IOP) and enables a greater number of measurements. Several studies have demonstrated its feasibility along with a high repeatability of measurements.
- The majority of patients with glaucoma can successfully measure their own IOP using the Icare HOME, after a 30-minute face-to-face training session and find the device easy to use.
- The COVID-19 pandemic has increased the demand for remote healthcare interactions and created an opportunity to change people’s attitude and experience toward them.

#### What this study adds

- Home tonometry can be taught via a video link with a success rate similar to previously reported rates for face-to-face teaching, using the same device. Those who successfully learnt how to use the device continued to take reliable measurements in their own home for 2 consecutive days.
- All participants were happy to be taught and found it easy to learn via the video link.
- This study offers the possibility of a true remote monitoring service for patients with glaucoma unable to attend their healthcare provider.
